# Long-Term Potable Effects of Alkalescent Mineral Water on Intestinal Microbiota Shift and Physical Conditioning

**DOI:** 10.1155/2019/2710587

**Published:** 2019-11-19

**Authors:** Takaaki Yahiro, Takao Hara, Takashi Matsumoto, Emi Ikebe, Nichole Fife-Koshinomi, Zhaojun Xu, Takahiro Hiratsuka, Hidekatsu Iha, Masafumi Inomata

**Affiliations:** ^1^Department of Microbiology, Oita University Faculty of Medicine, Oita, Japan; ^2^Department of Pathology, Tsurumi Hospital, Beppu, Oita, Japan; ^3^Department of Gastroenterological and Pediatric Surgery, Oita University Faculty of Medicine, Oita, Japan; ^4^Environmental Medicine Research Center, Quanzhou Medical College, Quanzhou, Fujian 362011, China

## Abstract

**Background:**

An alkalescent (pH 8.3) mineral water (AMW) of Hita basin, located in the northwestern part of Kyushu island in Japan, has been recognized for the unique quality of ingredients including highly concentrated silicic acid, sodium, potassium, and hydrogen carbonate. The biological effects of AMW intake were evaluated with a particular focus on its “antiobesity” properties through its modulation of the gut microbiota population.

**Methods:**

Two groups of C57BL6/J mice (8-week-old male) were maintained with a standard diet and tap water (control: TWC group) or AMW (AMW group) for 6 months and the following outputs were quantitated: (1) food and water intake, (2) body weight (weekly), (3) body fat measurements by CT scan (monthly), (4) sera biochemical values (TG, ALT, AST, and ALP), and (5) *UCP-1* mRNA in fat tissues (terminal point). Two groups of ICR mice (7-week-old male) were maintained with the same method and their feces were collected at the 0, 1st, 3rd, and 6th month at which time the population rates of gut microbiota were quantitated using metagenomic sequencing analysis of 16S-rRNA.

**Results:**

Among all antiobesity testing items, even though a weekly dietary consumption was increased (*p*=0.012), both ratios of weight gain (*p*=1.21*E* − 10) and visceral fat accumulation (*p*=0.029) were significantly reduced in the AMW group. Other criteria including water intake (*p*=0.727), the amounts of total (*p*=0.1602), and subcutaneous fat accumulation (*p*=0.052) were within the margin of error and *UCP-1* gene expression level (*p*=0.171) in the AMW group was 3.89-fold higher than that of TWC. Among 8 major gut bacteria families, Lactobacillaceae (increased, *p*=0.029) and Clostridiaceae (decreased, *p*=0.029) showed significant shift in the whole population.

**Conclusion:**

We observed significantly reduced (1) weight gaining ratio (average −1.86%, up to −3.3%), (2) visceral fat accumulation ratio (average −4.30%, up to −9.1%), and (3) changes in gut microbiota population. All these consequences could support the “health benefit” functionality of AMW.

## 1. Background

The importance of healthy fluid intake in children for physical and mental performance and health and in the prevention of obesity has been discussed and practiced [1–4]. Ingredients contained in mineral water for consumption such as magnesium [5, 6], calcium [7], or both [8] and bicarbonate [9] have demonstrated their ameliorating effects on metabolic diseases or health promoting functions through cohort studies in multiple locations worldwide. On the other hand, minerals such as nickel have parallel effects on body fat accumulation [10]. Since water must be consumed daily, the continuous intake of trace ingredients over a prolonged period could have certain effects on our physical conditions like those described above.

An alkalescent mineral water (AMW) produced from a ground-water artery beneath the Hita basin, located in the northwestern part of Kyushu island Japan, has been recognized for its distinct characteristic ingredients through filtration with the multiple volcanogeneous soil layers (at a depth of 750 m). AMW of Hita basin consists of significantly high concentrations of silicic acid (6.1-fold of the world average (WA) and 4.3-fold of the Japanese average (JA) of mineral water), potassium (5.0-fold of the WA and 10.0-fold of the JA), sodium (2.2-fold of the WA and 2.4-fold of the JA), and hydrogen carbonate (1.4-fold of the WA and 2.6-fold of the JA). On the other hand, AMW of Hita basin contains lower amounts of sulfates (3.0-fold lower than the WA and 2.8-fold lower than the JA), magnesium (2.0-fold lower than the WA), and calcium (1.6-fold lower than the WA). The combination of these ingredients contributes to making AMW an alkalescent (pH 8.3) and a soft water (see Supplemental [Supplementary-material supplementary-material-1]).

AMW has demonstrated its bioactive characteristics *in vitro*, aquaporin water permeability [11], immune cell activation [12], and protective effects on alloxan-induced pancreatic beta-cell damage through scavenging action against reactive oxygen species (ROS) [13]. Another aspect of AMW's function is to reduce anxiety. 180 consecutive days of AMW intake reduced the biomarkers of anxiety such as urinary 8-hydroxy-2′-deoxyguanosine and blood–urea nitrogen levels compared with the controls [14]. Therefore, these “health conditioning” functions may be attributed to the biologically active ingredients of AMW. In addition, antiobesity effects on mice fed a high-fat diet [15] and reduced blood viscosity in healthy adults [16] by AMW ingestion have also been reported. Shirahata et al. suggested that highly concentrated hydrogen carbonate could contribute in reducing ROS [13], which shares similar machinery with electrolyzed-reduced water (ERW) known to reduce intestinal abnormal fermentation, acid indigestion, chronic diarrhea, constipation, dyspepsia, and antacid [17]. Hydrogen-rich water also improves lipid and glucose metabolism in patients with type 2 diabetes or impaired glucose tolerance [18].

All of this evidence prompted us to test the possible effects of AMW on the population of gut microbiota (GM) and on antiobesity, which has been reported to have a close functional linkage between GM and gastrointestinal inflammation, metabolic diseases, and cognitive functions [19, 20]. Here we report the relevance between long-term ingestion of AMW and specific population control of GM and antiobesity function in mice.

## 2. Materials and Methods

### 2.1. Animals

**Figure 1:**
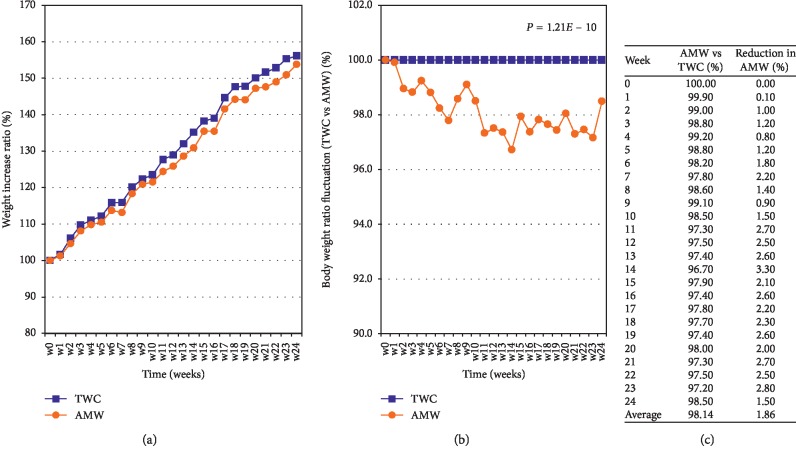
Comparison of weight gaining properties between TWC and AMW groups. (a) Average weight of each experimental group from weeks 0 to 24. The blue line represents TWC and the orange line represents AMW. Values were calculated from individual measurements of body weight. (b) The average body weights of both experimental groups at week 0 were defined as 100%, and the relative ratios from weeks 1 to 24. (c) The fluctuation of body weight ratios between TWC control and AMW test groups. The values of the control groups = 100% (blue line) throughout the experiments and the relative values of AMW (orange line) are shown. The statistical significance of each criterion was evaluated by Student's *t*-test and indicated as *p* values.

The experiments on mice were conducted as per our previous study [21]. Briefly, eight-week-old male C57BL6/J mice were fed a standard diet and kept under the specific-pathogen-free (SPF) conditions. Each group (*N* = 10) were fed a normal diet and given either tap water (TWC) for the control group or AMW for the test group to drink exclusively for 6 months (Supplemental [Supplementary-material supplementary-material-1]) [22]. The following outputs were quantitated: (1) food and water intake (weekly), (2) body weight (weekly), (3) body fat measurements by CAT scan (monthly), (4) biochemical values of sera (TG, ALT, AST, and ALP), and (5) *UCP-1* mRNA expression in fat tissues at the end of the study period (Supplemental [Supplementary-material supplementary-material-1]). To evaluate the relationship between antiobesity and gut microbiota, we employed the additional evaluation method previously described [23]. Three-week-old male ICR mice (Japan SLC, Inc., Shizuoka, Japan) were kept individually at 21 to 24°C and 30 to 60% humidity in a 12-hour light-dark cycle. They were given free access to TWC and standard laboratory chow. After 4-week acclimatization period, they were maintained with TWC or AMW (*N* = 4 each) in identical conditions as those shown in Supplemental [Supplementary-material supplementary-material-1] under the standard facility conditions (OPEN, Supplemental [Supplementary-material supplementary-material-1]). During the experimental period, TWC and AMW were exchanged to fresh ones every day, water bottles were autoclaved every week, and cages were disinfected with 70% ethanol every week. Individual body weights were monitored (Supplemental [Supplementary-material supplementary-material-1]) and their feces were collected at the 0, 1st, 3^rd^, and 6th month (M0, 1M, 3M, and 6M, hereafter) to identify the population rates of gut microbiota by 16S rRNA gene amplification and sequencing as described below. All mice related manipulations were performed with protocols approved by the animal ethics committee at the Oita University (Justified numbers, daily care, treatment, and euthanasia procedures). Statistical significance between AMW and TWC in Figure 1 was calculated by *R* version 3.5.1 “Feather Spray.”

### 2.2. Abdominal Computed Tomography (CT)

The visceral fat and subcutaneous fat of each mouse were measured using a RmCT2 3D micro X-ray CT scanner (Rigaku KK, Japan) once every month for six months. To keep the mice sedated before scanning, they were first anesthetized with isoflurane. Images were taken with a 20 *μ*m slice and the scanning duration for every mouse was 17 seconds.

### 2.3. Quantitation of Biochemical Values in Sera

At the end of the study, levels of triglycerides (TG), alkaline phosphatase (ALP), alanine aminotransferase (ALT), and aspartate aminotransferase (AST) were measured from mouse sera using A DRI-CHEM 4000 (Fuji Film KK, Japan). Results from both groups were compared and analyzed.

### 2.4. Quantitative Real-Time-Polymerase Chain Reaction (RT-PCR)

The expression levels of uncoupling protein-1 (UCP-1) were quantitated from the total RNA extracted from adipose tissue around the mice testicles using the Light-Cycler *R* 480 System (Roche, KK, Japan). Primer sequences and Universal Probe Library (UPL) Probe (Roche) number are listed in Supplemental [Supplementary-material supplementary-material-1].

### 2.5. DNA Extraction from Gut Fecal Samples

Fecal samples were frozen and dried using VD-250R Freeze Dryer (TAITEC Corp., Saitama, Japan) and were mechanically disrupted using Shake Master Neo (Bristol-Myers Squib. Co., Ltd., NY, USA). Total DNA was extracted using MPure Bacterial DNA Extraction Kit (MP Biomedicals Japan. Co., Ltd., Tokyo, Japan). The concentration of the extracted DNA was determined by using a Synergy H1 (Bio Tek, Instruments, Inc., Winooski, VT, USA) and QuantiFluor dsDNA System (Promega Corp., Madison, WI, USA), and samples were stored at −30°C until use.

### 2.6. 16S rRNA Gene Amplification and Sequencing

The V3-V4 region of bacterial 16S rRNA gene was amplified by using universal primers, 341F (5′-ACACTCTTTCCCTACACGACGCTCTTCCGATCT-CCTACGGGNGGCWGCAG-3′) and 805R (5′-GTGACTGGAGTTCAGACGTGTGCTCTTCCGATCTGACTACHVGGGTATCTAATCC-3′). PCR amplification was performed by using Ex Taq (TaKaRa Inc., Tokyo, Japan) with reaction at 94°C for 2 minutes, followed by 25 cycles at 94°C for 30 seconds, 55°C for 30 seconds, and 72°C for 30 seconds, with final elongation at 72°C for 5 minutes. Amplicons were purified by using Agencourt AMPure XP magnetic beads (Beckman Coulter, Brea, CA, USA). Unique indexes were added by using Nextera XT Index kit (Illumina Inc., San Diego, CA) with an additional 10 cycles of PCR. Following indexed PCR, DNA products were purified with 56.0 *μ*L of Agencourt AMPure XP (Beckman Coulter) and were eluted into 27.5 *μ*L of 10 mM Tris-HCl (pH 8.5), according to the manufacturer's protocol. The validation of the DNA library was done by a Fragment Analyzer system and dsDNA 915 Reagent kit (Advanced Analytical Technologies, Inc., Ankeny IA, USA). Finally, the pooled 5 pM of DNA library was denatured with 0.2 N of NaOH and mixed with PhiX Control v3 (Illumina Inc., San Diego, CA) at 15% of the final concentration as described in the Illumina procedure. Paired-end sequencing was conducted by next-generation sequencer MiSeq platform (Illumina Inc.) with MiSeq Reagent Kit version 3 (2 × 300 bp Paired-End Reads, Illumina Inc.)

### 2.7. 16S rRNA Sequence Data Analysis

Demultiplexing and fastq files generation were processed with the MiSeq Reporter Software (Illumina Inc). Reads were trimmed based on both their quality score and read length by Trimmomatic [24], and paired-end reads were generated by using PEAR. These output files were analyzed using downstream computational pipeline of the Quantitative Insights Into Microbial Ecology (QIIME) software package version 1.9.0. Chimeric reads were filtered using UCHIME, and sequences were assigned to operational taxonomic units (OTU) using the QIIME implementation of UCLUST with an OTU cluster defined as a sequence similarity of 97%. Bacterial taxonomy was assigned with the uclust consensus taxonomy assigner against the Greengenes database v13_8 clustered at 97%. OTUs biom table was rarefied by the single_rarefaction.py script to make random subsampling on the basis of a minimum rarefaction depth value at 25,096 sequences/sample. Entire results of the metagenome sequencing analysis will be supplied upon request.

## 3. Results

### 3.1. Effects of Alkalescent Mineral Water (AMW) Intake on Body Weight Increase in Mice

To evaluate the antiobesity effects of AMW, we first divided 8-week-old C57BL/6 mice into two groups, the tap water control (TWC) group and test alkalescent mineral water (AMW) group, and they were maintained for 6 months under the specific-pathogen-free (SPF) conditions. Individual diet consumption, water intake, and change in body weight were measured and recorded every week (Supplemental [Supplementary-material supplementary-material-1]).

**Figure 2:**
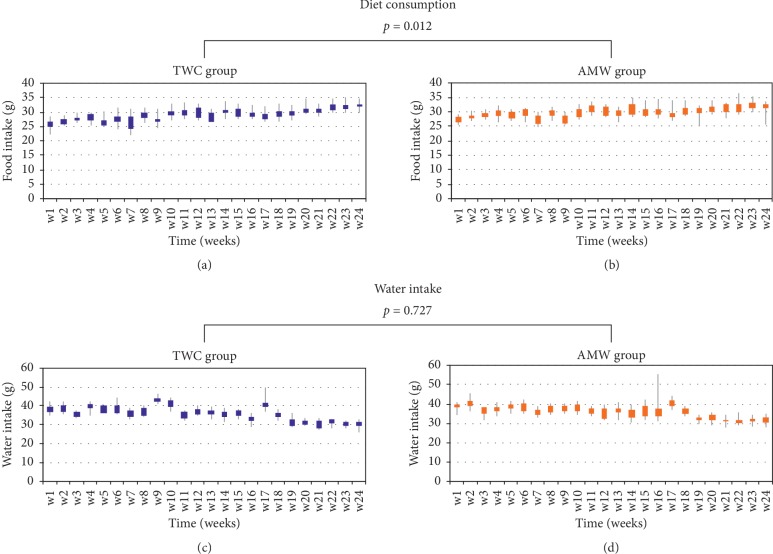
Fluctuation of weekly diet and water consumption in either TWC or AMW ingestion group. The average amount of diet consumed each week was calculated for each group of C57BL6/J mice (*N* = 10, kept in SPF conditions, see Section 2). Diet consumption of the control TWC (a) and the testing AMW groups (b), or water intake in TWC (c) and AMW groups (d). The statistical significance of each criterion was evaluated by ANOVA and indicated as *p* values.

Results for the average amounts of weekly diet consumption and water intake of both TWC and AMW groups are shown in Figure 2. The average dietary consumption for AMW, 29.9 g (SD: 2.1 g, Figure 2(b)), significantly exceeded (3.8% increase, *p*=0.012) the values from TWC, 28.8 g (SD: 2.5 g, Figure 2(a)). On the other hand, the average water intake for both did not show any difference (*p*=0.727, TWC: 36.1 mL (SD: 4.2 mL) and AMW: 36.0 mL (SD: 3.7 mL); Figures 2(c) and 2(d)).

Individual body weights were monitored weekly, the average weight gaining ratio for AMW fell below those of TWC throughout the 6 month's observation periods (Figure 1(a)), and the average reduction rate was −1.86% (Figure 1(b), *p*=1.21*E* − 10). The detailed values are displayed in Figure 1(c).

### 3.2. AMW Intake Effects on Abdominal Adipose Tissue Accumulation

**Figure 3:**
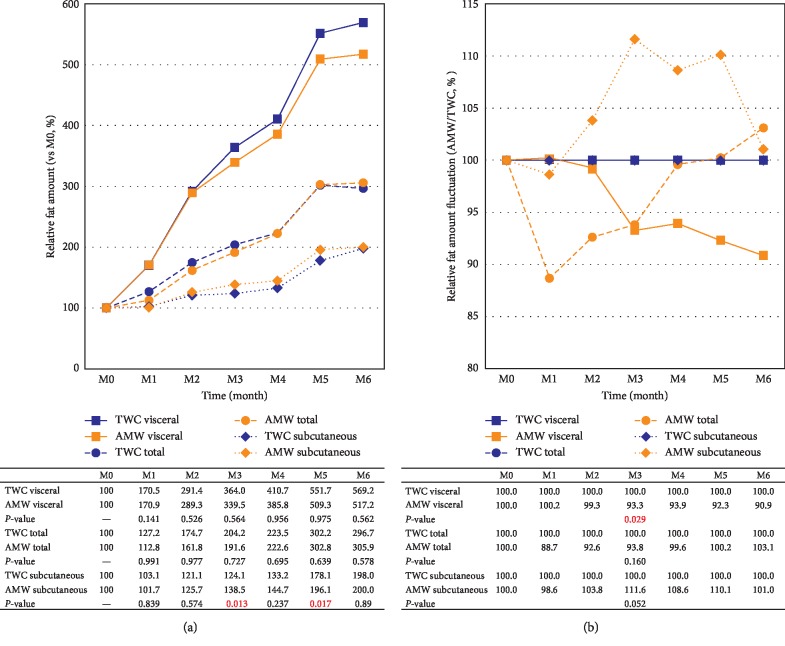
Effects of AMW intake on body fat fluctuation. (a) Average amounts of visceral fat (solid lines with square markers), total fat (dotted lines with circle markers), and subcutaneous fat (dotted lines with diamond markers) of the six experimental groups at week 0 = 100%, and the relative ratios at every month are shown. (b) The fluctuation of body fat ratios between TWC control and AMW test group. The values of the control groups = 100% (blue line) throughout the experiments and the relative values of AMW (orange line) are shown. The statistical significance of each criterion was evaluated by ANOVA and indicated as *p* values in each lower panel.

Abdominal fat mass determination by CT scan was performed on each mouse once a month for the whole duration of the study (Supplemental [Supplementary-material supplementary-material-1]) to measure the visceral and subcutaneous fat (Figure 3(a)) and revealed a significant decrease of visceral fat in the AMW group (Figure 3(b), *p*=0.029). On the other hand, subcutaneous fat seemed to be accumulated more in the AMW group (*p*=0.052).

Upon dissection of the test subjects at the end of the study, adipose tissues from around the mice testicles were harvested during dissection and the total RNA was extracted from them. Gene expression levels of the *UCP-1* gene were measured using quantitative RT-PCR. The AMW group mice expressed the *UCP-1* gene 3.89 times more (*p*=0.171) than those of TWC (Supplemental [Supplementary-material supplementary-material-1]). Again, this result suggests a possible machinery in AMW that could induce *UCP-1* expression in beige adipocytes.

We also evaluated the biochemical values related to obesity in sera, such as TG, ALP, ALT, or AST at the terminal point but no significant difference was detected (Supplemental [Supplementary-material supplementary-material-1]).

### 3.3. Ingestion of Alkalescent Mineral Water Results in a Shift in Gut Microbiota

Since we suspected that the antiobesity function of AMW was partly the result of gut microbiota fluctuation, we conducted metagenome sequencing analysis of the gut microbiota under the following conditions (Supplemental [Supplementary-material supplementary-material-1]). First, we changed the rearing environment of mice from clean specific-pathogen-free (SPF) to normal facility (OPEN) conditions; second, we followed the mice microbiota evaluation system according to the formally established method that utilizes ICR strain instead of C57BL6 [23]. Two groups of male ICR mice were maintained in the same conditions and given either AMW or TWC. We have monitored the amount of drinking water, diet consumption (data not shown), and body weight every week (Supplemental [Supplementary-material supplementary-material-1]). Although we did not observe any significant differences in all three antiobesity criteria, body weight increase ratio of the AMW group seemingly went lower than that of control at the last 6 weeks of the experiment (Supplemental Figures [Supplementary-material supplementary-material-1] and [Supplementary-material supplementary-material-1]).

**Figure 4:**
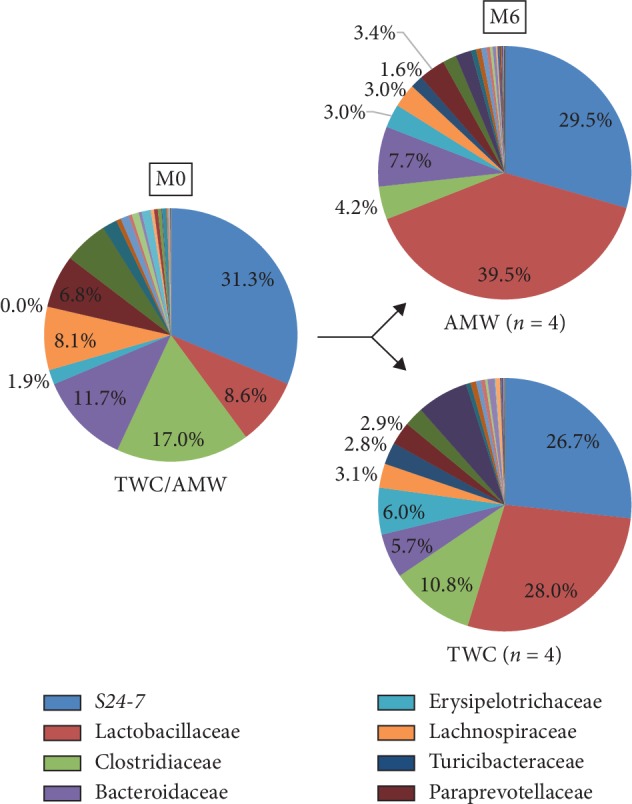
Comparison of fluctuation in properties of gut microbiota population between TWC and AMW groups. Feces collected from ICR mice in both TWC and AMW groups were analyzed with metagenomic sequencing analysis of 16S-rRNA as indicated in Section 2. Proportions of each bacterium at the level of family identified at M0 and M6 are shown in pie charts.

**Figure 5:**
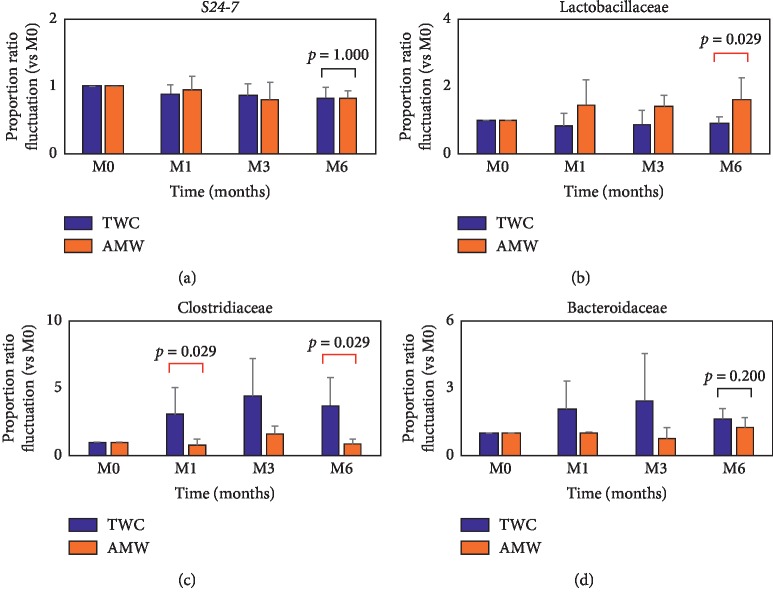
Significant shift of gut flora population in mice after continuous supplementation of TWC and AMW for 6 months. Fluctuation in the population ratio of four major families of gut flora was calculated at each time point as listed. The statistical significance of each criterion was evaluated by Mann–Whitney *U* test and indicated as *p* values.

Then the feces of both groups were collected at M0, M1, M3, and M6 and the family groups of gut microbiomes were determined at each time point by metagenomic sequencing analysis of 16SrRNA (see Section 2). Of the total 92 bacteria families detected at the beginning of the experiment, the top eight families occupied 85% of the whole population (Figure 4, indicated as M0). After six months of ingesting AMW or TWC, a shift in the population of gut microbiome was found as indicated in the two graphs (Figure 4, indicated as M6, respectively). From these results, the fluctuation of four families' population in their gut flora was displayed (Figure 5). While almost stable behavior was observed in *S24-7* (*p*=1.000, Figure 5(a)), an increasing tendency with Lactobacillaceae (*p*=0.029, Figure 5(b)) and decreasing tendency with Clostridiaceae (*p*=0.029, Figure 5(c)) in the AMW group were observed. Bacteroidaceae population was also decreasing in AMW without statistical significance anyway (*p*=0.200, Figure 5(d)). Details of individual population shift (Supplement [Supplementary-material supplementary-material-1]) and fluctuation ratios (Supplement [Supplementary-material supplementary-material-1]) were summarized.

## 4. Discussion

AMW of Hita basin has been characterized for containing various minerals (Supplemental [Supplementary-material supplementary-material-1]). Although the amount of trace ingredients or minerals in the drinking water are significantly variable from place to place even in Japan, the average values of major ingredients are summarized in Supplemental [Supplementary-material supplementary-material-1] [22, 25]. Distinctive characteristics of AMW are clearly recognizable: high silicic acid, potassium, hydrogen, and carbonate; and low magnesium, calcium, and sulfate. Since previous reports have already described the significantly different biological outputs after the long-term intake of mineral water to distilled water intake controls [13, 14, 22, 26], we evaluated the biological function of AMW by comparing with TWC but not with distilled water. To date, several studies have shown AMW's scavenging action against reactive oxygen species (ROS) to protect cells from different stress conditions such as DNA damage and inflammation [13, 14]. The ROS scavenging property of the “active hydrogen” in water has been linked to the suppression of tumor angiogenesis in mice [27].

In our study, the antiobesity effects of AMW with significant increase in diet intake (Figure 2) and suppressed weight gaining ratio (1.86% less than those of TWC) were observed in the AMW group (Figure 1). We also observed significant (*p*=0.029) reduction of visceral fat (−4.30% average, Figure 3). Although there was no statistical significance, 3.89-fold induction of *UCP-1* gene expression in the adipose tissue (Supplemental [Supplementary-material supplementary-material-1]) in AMW group was also observed. *UCP-1* is exclusively found in the brown adipose tissues whose primary function has been associated with the production of heat [28, 29]. Increased expression of the *UCP-1* gene has also been associated with obesity control and insulin insensitivity in mice [29]. Serum biomarkers for obesity or liver dysfunction indicated no significant differences as well (Supplemental [Supplementary-material supplementary-material-1]).

Collected results in this study seemed to reflect the evidence in clinical surveys linking obesity (or metabolic diseases) to gut microbiota fluctuation [30, 31] or an animal model system suggesting a linkage between gut microbiota alteration caused by drinking water and amelioration of type 1 diabetes symptoms [32].

While these collective results strongly implied certain antiobesity effects with AMW, we had some technical concerns on the obtained results as follows: (1) mice were kept in “too clean” SPF environment which does not reflect our daily life and (2) an inbred C57BL6 is known to display a “Th1 oriented” immune response properties [33, 34] and this T-cell-type immune variations is known to affect significantly the microbiota population [34, 35]. Therefore, our next approach was to prove the long-term intake effects of AMW on antiobesity functionality by selecting a closed colony strain ICR and we have already established our own experimental procedures with conventional rearing environment [23]. We collected feces from mice at the beginning (M0) and after 1, 3, and 6 months feeding of either with TWC or AMW (M1, M3, and M6) to analyze the gut microbiota population by metagenomic sequencing analysis of 16S-rRNA (Supplemental [Supplementary-material supplementary-material-1]). S24-7 family which is a bacterium belonging to phylum Bacteroidetes and known to be a predominant member of the gut microbiota in animals [36] did not show any significant difference in its proportion by TWC or AMW intake (Figure 5(a)). Among the eight major populations of gut microbiota detected in this study, statistically significant fluctuations of Lactobacillaceae and Clostridiaceae were observed (Figures 4 and 5). *Lactobacillus spp*. has been known as one of the probiotic species which ameliorates metabolic disorders in human [37, 38]. With regard to Clostridiaceae, judgement is rather difficult. While the deteriorating effects of *Clostridia spp*., such as *C. difficile* on gut microbiota, is evident [39], the beneficial function of *C. butyricum* has been well established [40]. However, most of *Clostridia* spp. can be categorized as pathogenic. In this study, we collected stool samples from 4 individual mice kept in a rigidly controlled laboratory environment according to the previous gut microbiota studies [41–44]. The symbiotic relationship between human hosts and gut microbiota benefits us in many ways. One of the most important of which is it allows us to extract calories from the indigestible polysaccharides in our diet and, through a pathway regulated by these bacteria, store this harvested energy in our adipocytes [45, 46]. The two most dominant populations of bacteria in the gastrointestinal tract of both humans are from the gram-negative phylum Bacteroidetes and gram-positive phylum Firmicutes which includes the class Clostridia [47]. In both studies on humans and mice, it was found that the ratio of these 2 dominant populations of bacteria in the gut is associated with obesity. Firmicutes was found to be significantly increased in mice and human subjects of the study that were classified as obese and the Bacteroidetes was decreased [45–48]. It is therefore premature to attribute our observation of AMW's antiobesity activity to the proportional fluctuation of the Lactobacillaceae and Clostridiaceae. However, *Lactobacillus* spp. is known to be one of the major bacterial species used as probiotics and is believed to improve digestive health. In studies on mice administrated with *Lactobacillus* spp., it was found that it aided in the reduction of body weight and fat accumulation which resulted in a positive outcome on lipid, insulin, and liver toxicity biomarker levels [49–51]. The linkage between gut microbiota fluctuation and multiple biological effects including obesity, tumorigenesis, and mental disorder has already been investigated [29, 30, 52, 53]. The exact molecular mechanism of the present study is yet to be elucidated; however, our approach to evaluate the biological effects of water ingestion could be expanded. We are conducting additional experiment programs to gain statistically reliable results. It has been reported that rats which have ingested AMW for 6 months displayed a more active behavior as compared to those that were only given TWC [14]. This may account for the weight gain suppression observed in mice despite the increase in food and water intake, as well as the reduction in fat mass measured.

## 5. Conclusion

We demonstrated here the long-term potable effects of AMW on the population fluctuation of gut microbiota for the first time. Six-month intake of AMW induced the increase of typical probiotic Lactobacillaceae and the decrease of mostly unfavorable Clostridiaceae. This gut microbiota fluctuation by AMW intake was seemingly linked with a tendency of antiobesity effects. Since we drink water on daily basis, a certain combination of ingredients in drinking water (as demonstrated in this study) is expected to contribute to any adjunctive antiobesity/anti-inflammatory effects in a very moderate manner. Further molecular biological studies are scheduled to examine how AMW affects the gut microbiota population.

## Data Availability

We included all the data to assess the integrity of the whole experiments.

## Abbreviations

AMW: Alkalescent mineral water
UCP-1: Uncoupled protein-1
CT scan: Computed tomography scan
TWC: Tap water control
ROS: Reactive oxygen species
TG: Triglycerides
ALP: Alkaline phosphatase
ALT: Alanine aminotransferase
AST: Aspartate aminotransferase.

## Supplementary Materials

Supplementary Materials Supplementary Figure 1: comparison of the average major ingredients composition of mineral water among alkalescent mineral water (AMW) of Hita basin and those of global or Japanese areas. Supplementary Figure 2: schematic diagram of the diet study. Supplementary Figure 3: induction of UCP-1 gene expression in the testicle adipose tissues by AMW supplementation. Supplementary Figure 4: schematic diagram of the microbiota study. Supplementary Figure 5: comparison of weight gaining properties between TWC and AMW groups. Supplemental Table 1: comparison of major ingredients between TWC and AMW of Hita basin. Supplemental Table 2: primers and UPL probes used for RT-qPCR. Supplemental Table 3: average biochemical values in sera of C57BL6/J mice supplemented with TWC and AMW. Supplemental Table 4: gut microbiota population shift during 6-month intake of TWC and AMW. Supplemental Table 5: fluctuation ratio of four populations of gut microbiota and their standard errors (A) and standard deviations (B).
