# Corrigendum to “Long-Term Potable Effects of Alkalescent Mineral Water on Intestinal Microbiota Shift and Physical Conditioning”

**DOI:** 10.1155/2020/6706239

**Published:** 2020-10-05

**Authors:** Takaaki Yahiro, Takao Hara, Takashi Matsumoto, Emi Ikebe, Nichole Fife-Koshinomi, Zhaojun Xu, Takahiro Hiratsuka, Hidekatsu Iha, Masafumi Inomata

**Affiliations:** ^1^Department of Microbiology, Oita University Faculty of Medicine, Oita, Japan; ^2^Department of Pathology, Tsurumi Hospital, Beppu, Oita, Japan; ^3^Department of Gastroenterological and Pediatric Surgery, Oita University Faculty of Medicine, Oita, Japan; ^4^Environmental Medicine Research Center, Quanzhou Medical College, Quanzhou, Fujian 362011, China

In the article titled “Long-Term Potable Effects of Alkalescent Mineral Water on Intestinal Microbiota Shift and Physical Conditioning” [1], information was omitted in the Ethical Approval section. The corrected section appears below.
