# Supplementary material for: Long-Term Potable Effects of Alkalescent Mineral Water on Intestinal Microbiota Shift and Physical Conditioning
Source: Evid Based Complement Alternat Med. 2019 Nov 19;2019:2710587. doi: 10.1155/2019/2710587 (PMC6885775; doi:10.1155/2019/2710587)
Supplement: Supplementary Materials — Supplementary Figure 1: comparison of the average major ingredients composition of mineral water among alkalescent mineral water (AMW) of Hita basin and those of global or Japanese areas. Supplementary Figure 2: schematic diagram of the diet study. Supplementary Figure 3: induction of UCP-1 gene expression in the testicle adipose tissues by AMW supplementation. Supplementary Figure 4: schematic diagram of the microbiota study. Supplementary Figure 5: comparison of weight gaining properties between TWC and AMW groups. Supplemental Table 1: comparison of major ingredients between TWC and AMW of Hita basin. Supplemental Table 2: primers and UPL probes used for RT-qPCR. Supplemental Table 3: average biochemical values in sera of C57BL6/J mice supplemented with TWC and AMW. Supplemental Table 4: gut microbiota population shift during 6-month intake of TWC and AMW. Supplemental Table 5: fluctuation ratio of four populations of gut microbiota and their standard errors (A) and standard deviations (B). [file 2710587.f1.pptx]

## Slide 1
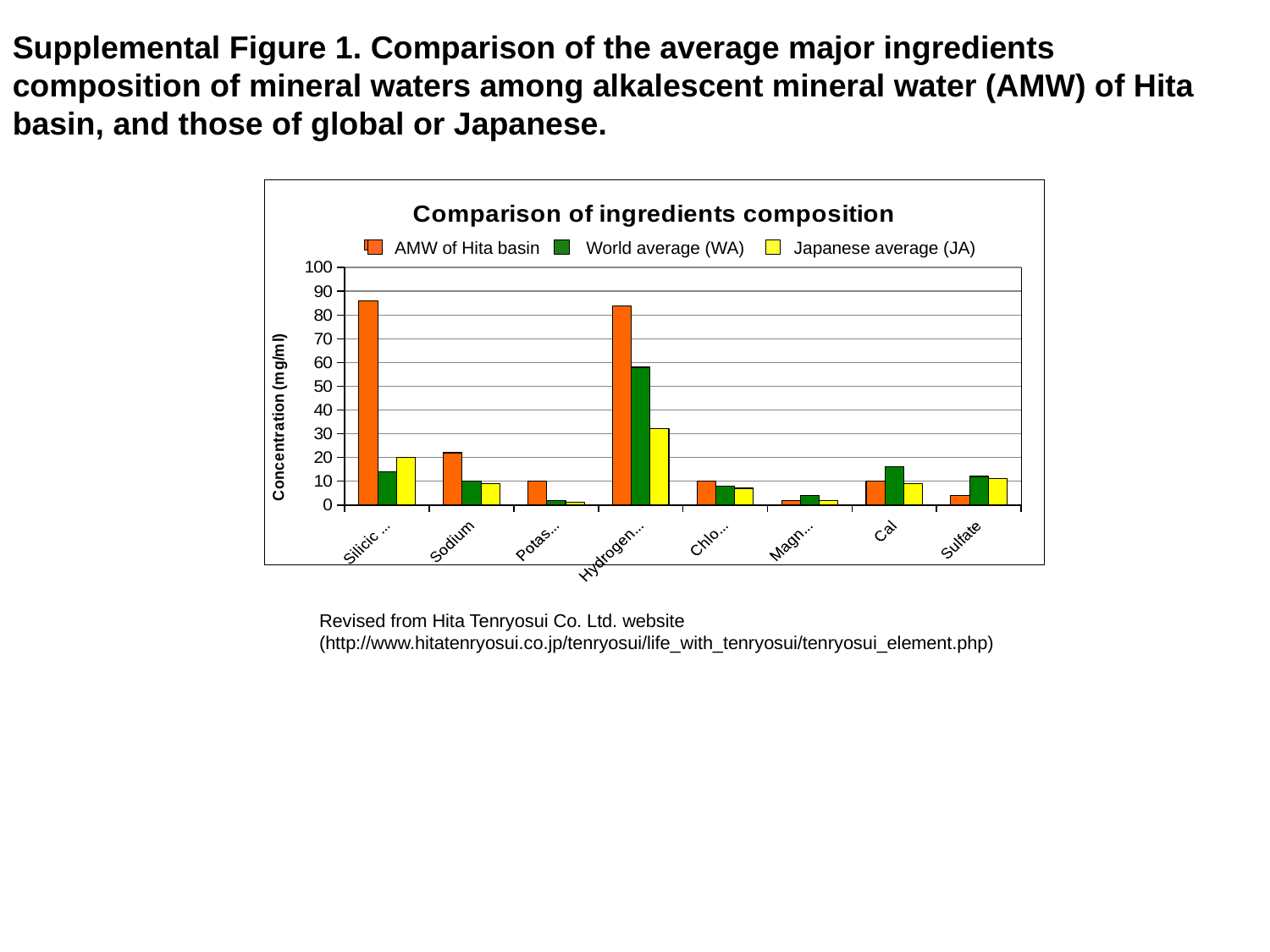

Supplemental Figure 1. Comparison of the average major ingredients composition of mineral waters among alkalescent mineral water (AMW) of Hita basin, and those of global or Japanese.
### Chart: Comparison of ingredients composition
| Category | Alkarescent Hita Water | World Average (WA) | Japan Average (JA) |
|---|---|---|---|
| Silicic acid | 86.0 | 14.0 | 20.0 |
| Sodium | 22.0 | 10.0 | 9.0 |
| Potassium | 10.0 | 2.0 | 1.0 |
| Hydrogen carbonate | 84.0 | 58.0 | 32.0 |
| Chloride | 10.0 | 8.0 | 7.0 |
| Magnesium | 2.0 | 4.0 | 2.0 |
| Calcium | 10.0 | 16.0 | 9.0 |
| Sulfate | 4.0 | 12.0 | 11.0 |
AMW of Hita basin
World average (WA)
Japanese average (JA)
Revised from Hita Tenryosui Co. Ltd. website
(http://www.hitatenryosui.co.jp/tenryosui/life_with_tenryosui/tenryosui_element.php)

## Slide 2
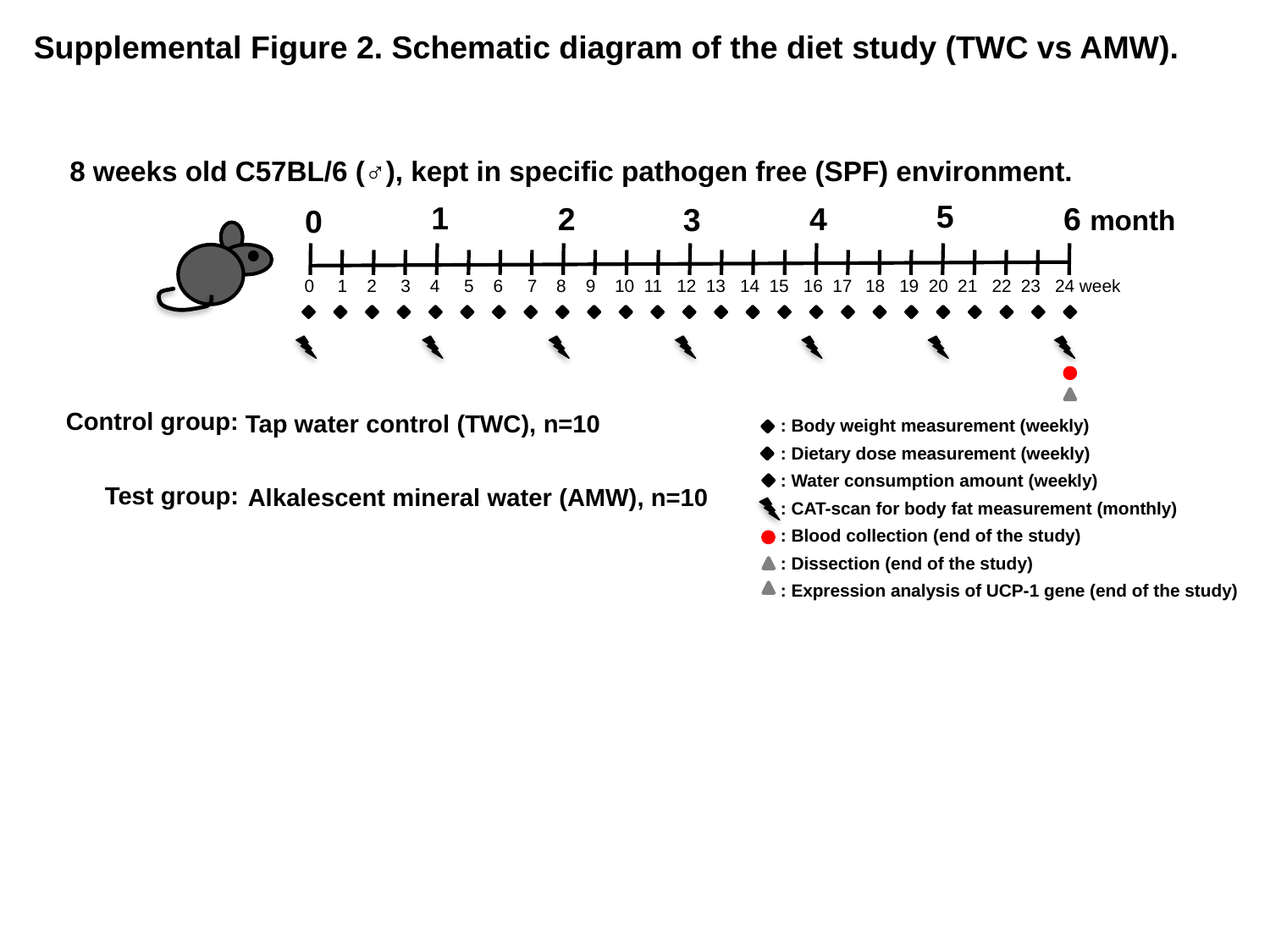

Supplemental Figure 2. Schematic diagram of the diet study (TWC vs AMW).
8 weeks old C57BL/6 (♂), kept in specific pathogen free (SPF) environment.
5
1
6 month
4
2
3
0
0 1 2 3 4 5 6 7 8 9 10 11 12 13 14 15 16 17 18 19 20 21 22 23 24 week
Control group:
Tap water control (TWC), n=10
: Body weight measurement (weekly)
: Dietary dose measurement (weekly)
: Water consumption amount (weekly)
: CAT-scan for body fat measurement (monthly)
: Blood collection (end of the study)
: Dissection (end of the study)
: Expression analysis of UCP-1 gene (end of the study)
Test group:
Alkalescent mineral water (AMW), n=10

## Slide 3
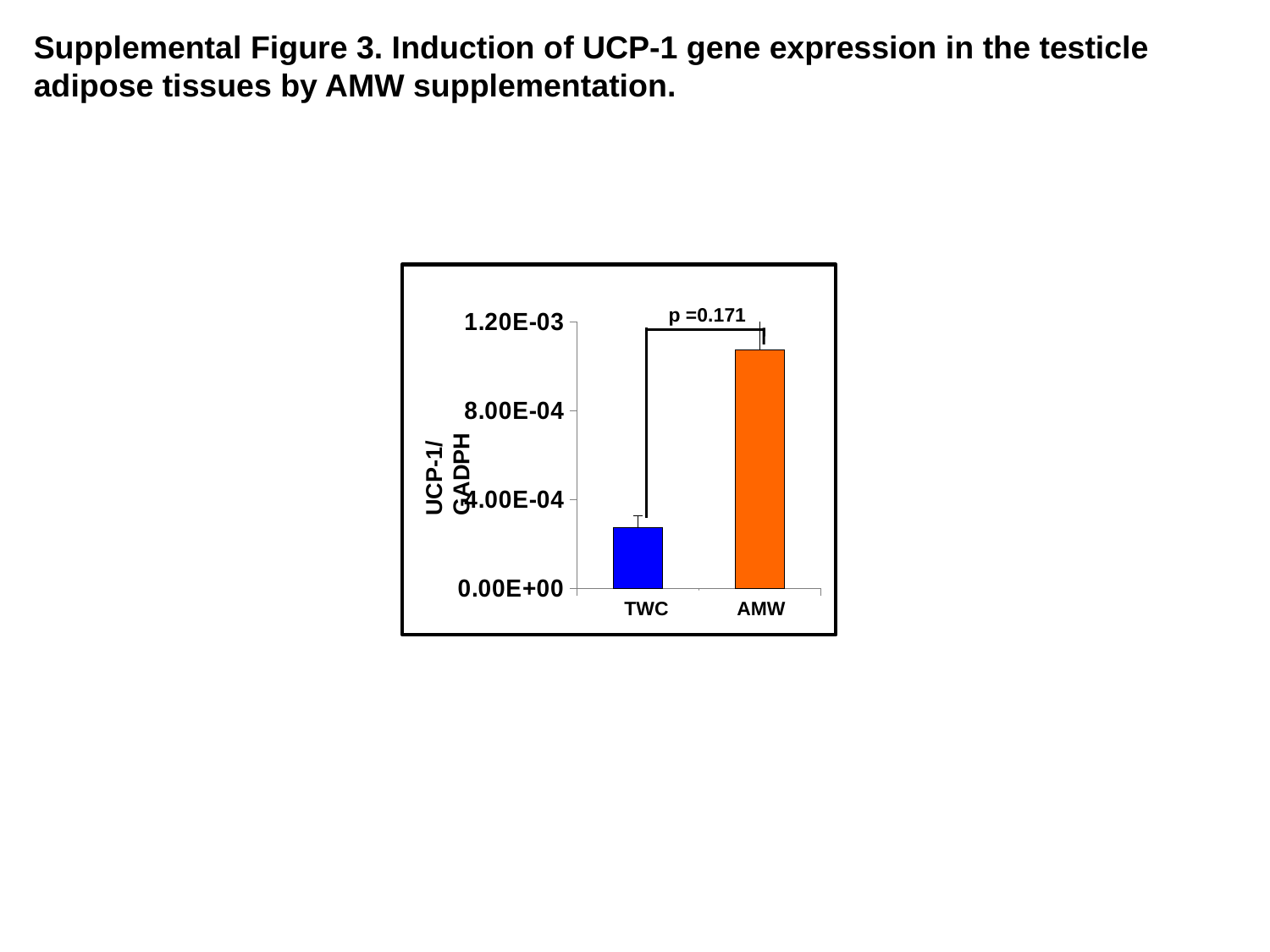

Supplemental Figure 3. Induction of UCP-1 gene expression in the testicle adipose tissues by AMW supplementation.
### Chart
| Category | |
|---|---|
| 水道水 | 0.000274861111111113 |
| 日田天領水 | 0.00107066944444444 |UCP-1/GADPH
TWC
 AMW
p =0.171

## Slide 4
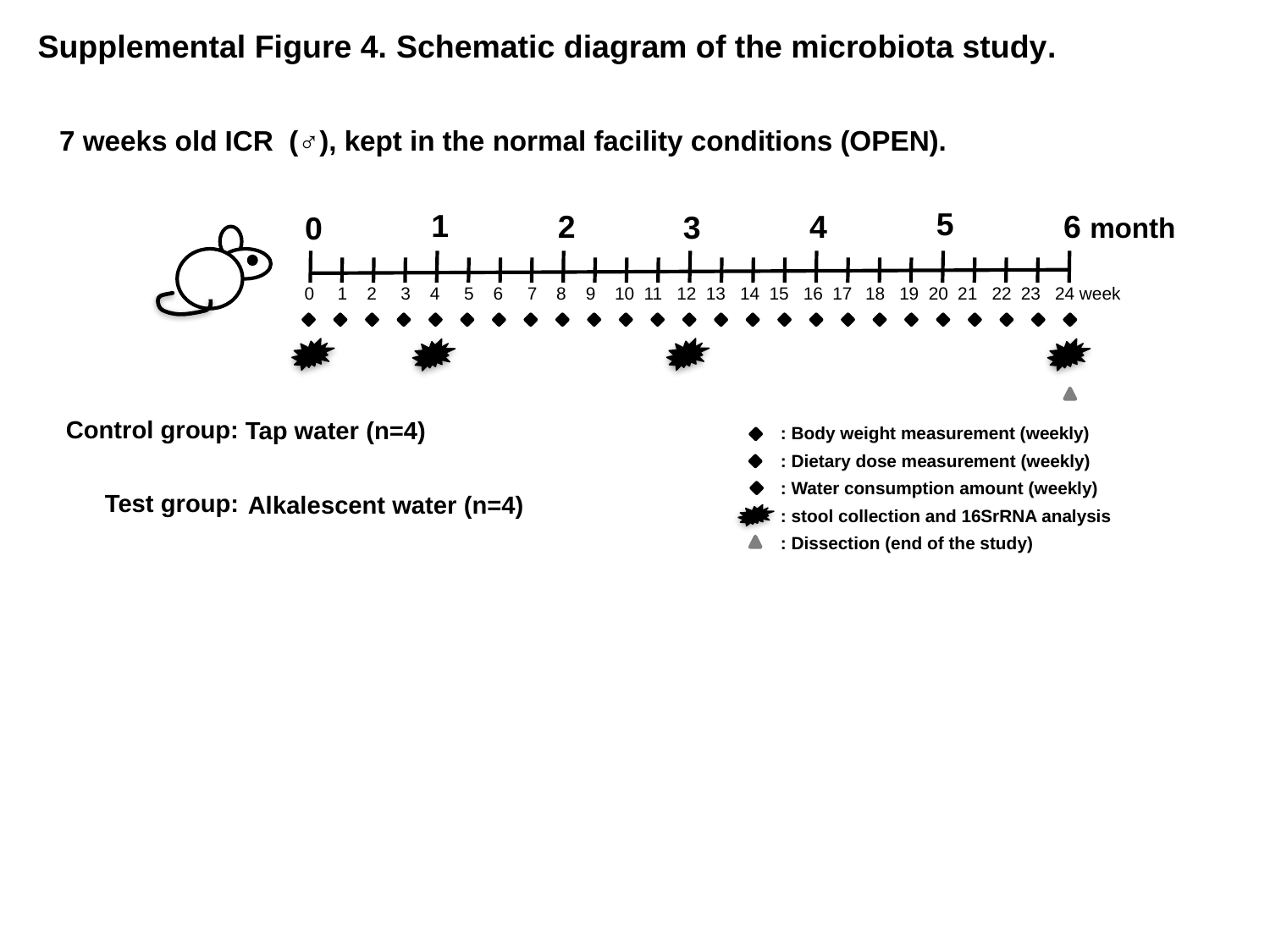

Supplemental Figure 4. Schematic diagram of the microbiota study.
7 weeks old ICR (♂), kept in the normal facility conditions (OPEN).
5
1
6 month
4
2
3
0
0 1 2 3 4 5 6 7 8 9 10 11 12 13 14 15 16 17 18 19 20 21 22 23 24 week
Control group:
Tap water (n=4)
: Body weight measurement (weekly)
: Dietary dose measurement (weekly)
: Water consumption amount (weekly)
: stool collection and 16SrRNA analysis
: Dissection (end of the study)
Test group:
Alkalescent water (n=4)

## Slide 5
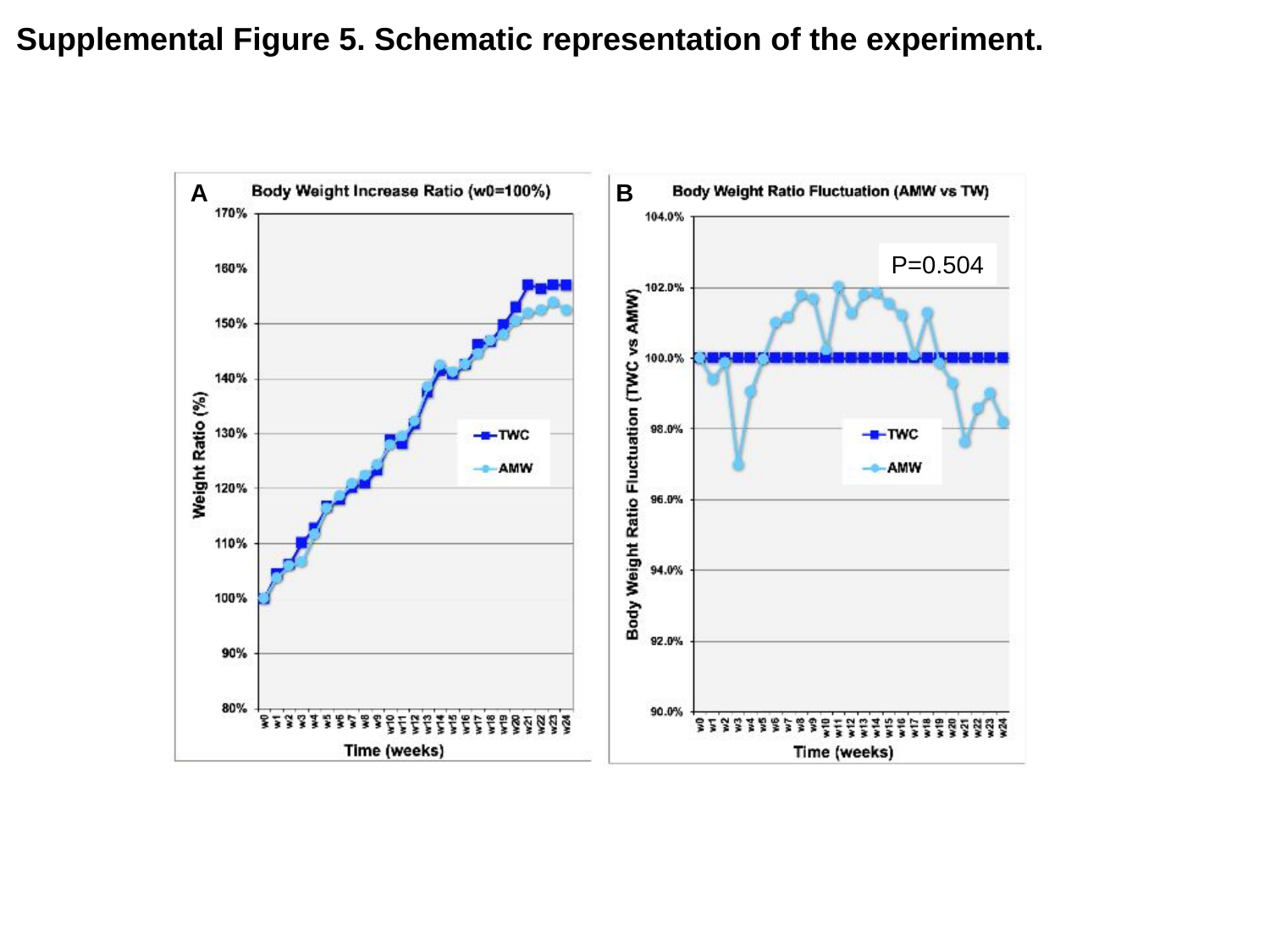

Supplemental Figure 5. Schematic representation of the experiment.
A
B
P=0.504

## Slide 6
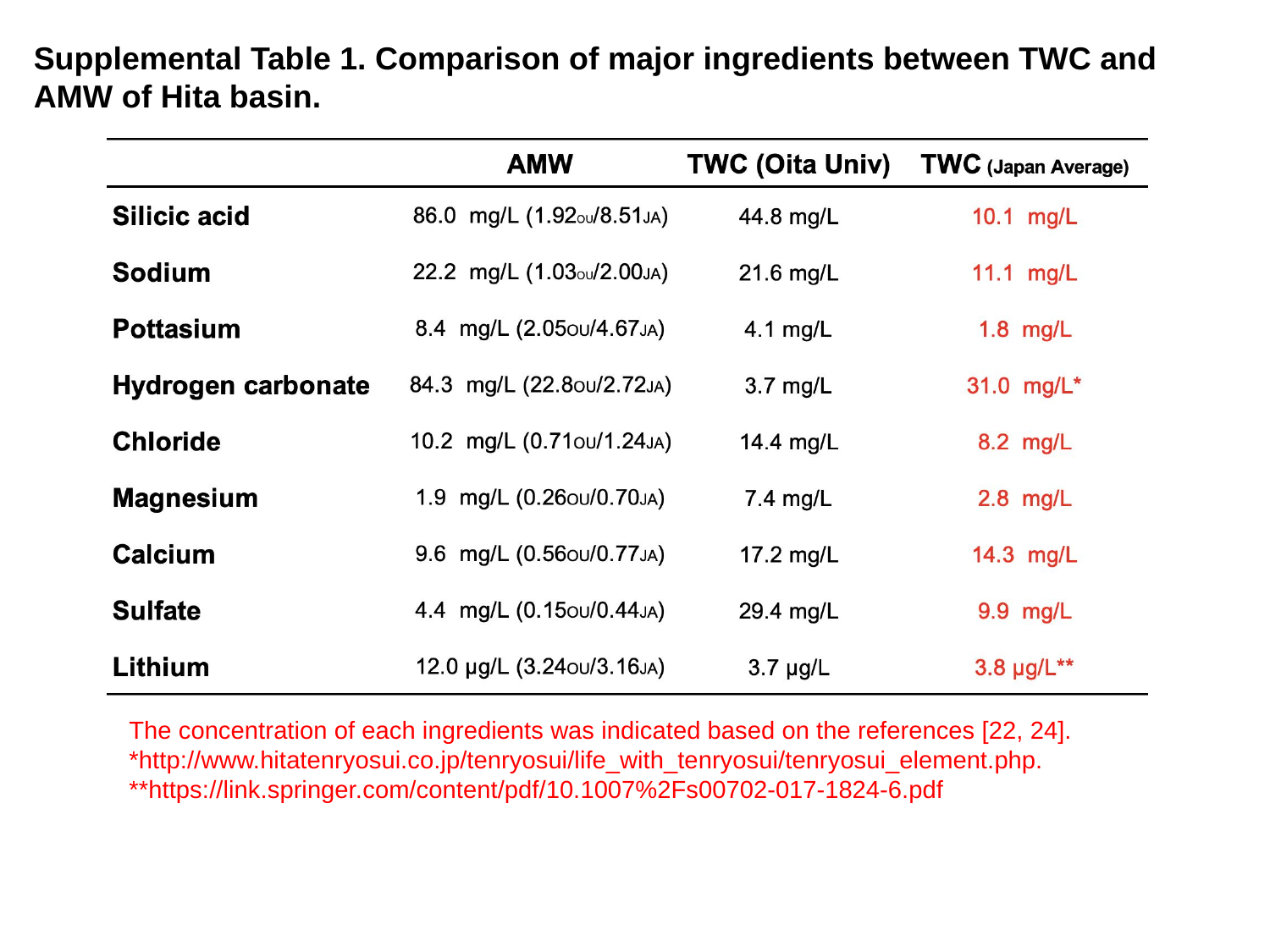

Supplemental Table 1. Comparison of major ingredients between TWC and AMW of Hita basin.
The concentration of each ingredients was indicated based on the references [22, 24].
*http://www.hitatenryosui.co.jp/tenryosui/life_with_tenryosui/tenryosui_element.php.
**https://link.springer.com/content/pdf/10.1007%2Fs00702-017-1824-6.pdf

## Slide 7
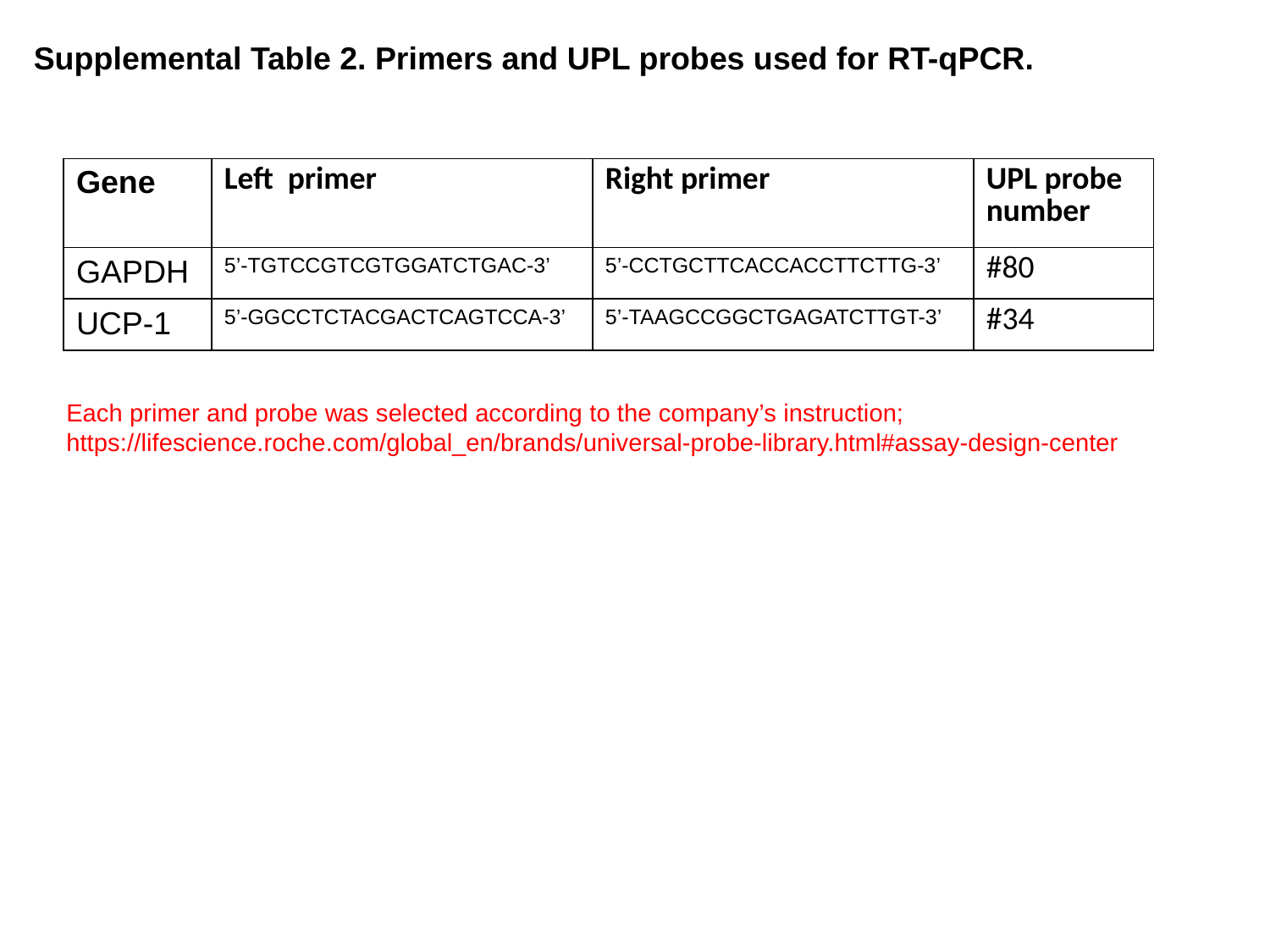

Supplemental Table 2. Primers and UPL probes used for RT-qPCR.
| Gene | Left primer | Right primer | UPL probe number |
| --- | --- | --- | --- |
| GAPDH | 5’-tgtccgtcgtggatctgac-3’ | 5’-cctgcttcaccaccttcttg-3’ | #80 |
| UCP-1 | 5’-ggcctctacgactcagtcca-3’ | 5’-taagccggctgagatcttgt-3’ | #34 |
Each primer and probe was selected according to the company’s instruction;
https://lifescience.roche.com/global_en/brands/universal-probe-library.html#assay-design-center

## Slide 8
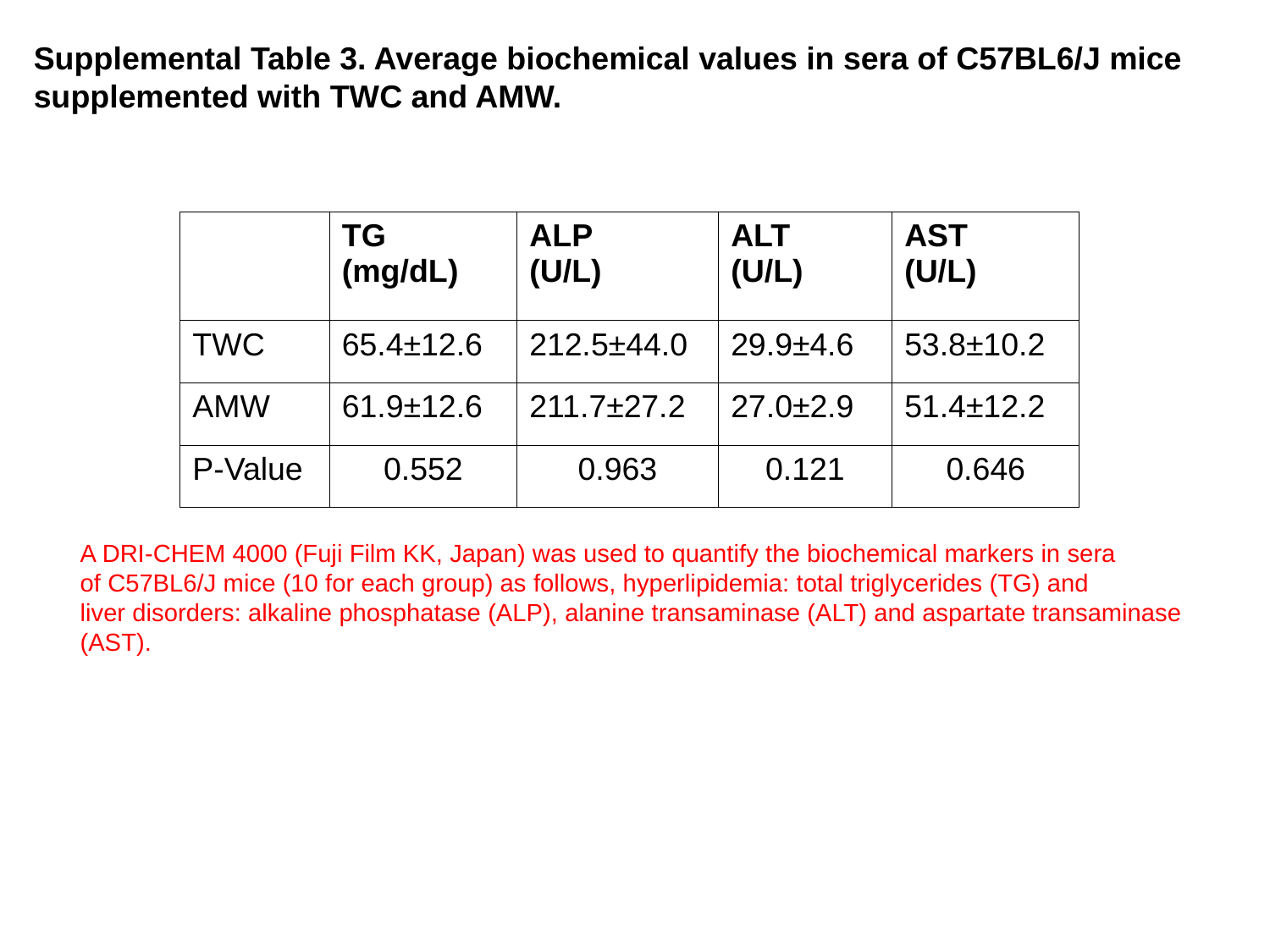

Supplemental Table 3. Average biochemical values in sera of C57BL6/J mice supplemented with TWC and AMW.
| | TG (mg/dL) | ALP (U/L) | ALT (U/L) | AST (U/L) |
| --- | --- | --- | --- | --- |
| TWC | 65.4±12.6 | 212.5±44.0 | 29.9±4.6 | 53.8±10.2 |
| AMW | 61.9±12.6 | 211.7±27.2 | 27.0±2.9 | 51.4±12.2 |
| P-Value | 0.552 | 0.963 | 0.121 | 0.646 |
A DRI-CHEM 4000 (Fuji Film KK, Japan) was used to quantify the biochemical markers in sera
of C57BL6/J mice (10 for each group) as follows, hyperlipidemia: total triglycerides (TG) and
liver disorders: alkaline phosphatase (ALP), alanine transaminase (ALT) and aspartate transaminase
(AST).

## Slide 9
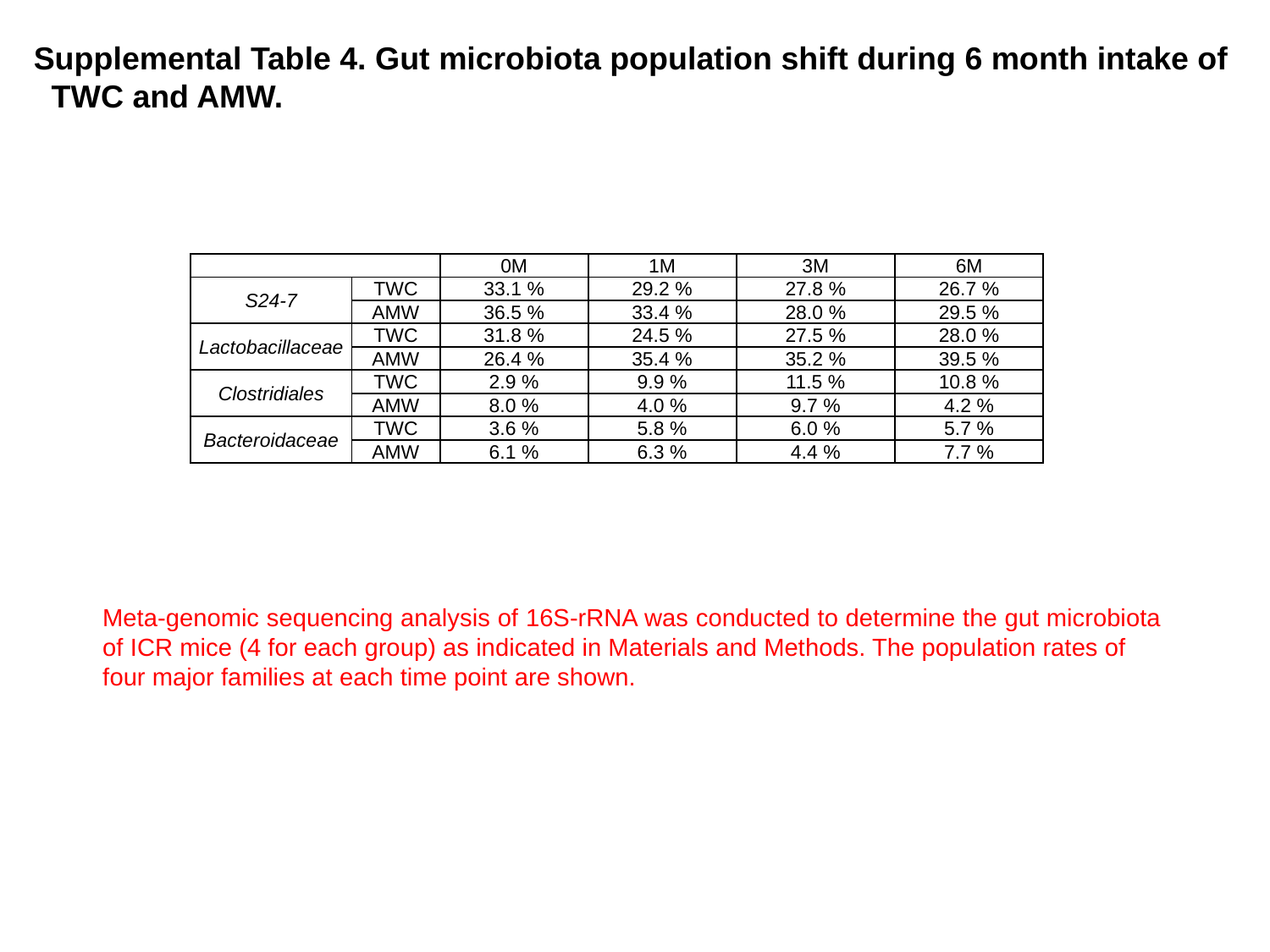

Supplemental Table 4. Gut microbiota population shift during 6 month intake of TWC and AMW.
| | | 0M | 1M | 3M | 6M |
| --- | --- | --- | --- | --- | --- |
| S24-7 | TWC | 33.1 % | 29.2 % | 27.8 % | 26.7 % |
| | AMW | 36.5 % | 33.4 % | 28.0 % | 29.5 % |
| Lactobacillaceae | TWC | 31.8 % | 24.5 % | 27.5 % | 28.0 % |
| | AMW | 26.4 % | 35.4 % | 35.2 % | 39.5 % |
| Clostridiales | TWC | 2.9 % | 9.9 % | 11.5 % | 10.8 % |
| | AMW | 8.0 % | 4.0 % | 9.7 % | 4.2 % |
| Bacteroidaceae | TWC | 3.6 % | 5.8 % | 6.0 % | 5.7 % |
| | AMW | 6.1 % | 6.3 % | 4.4 % | 7.7 % |
Meta-genomic sequencing analysis of 16S-rRNA was conducted to determine the gut microbiota
of ICR mice (4 for each group) as indicated in Materials and Methods. The population rates of
four major families at each time point are shown.

## Slide 10
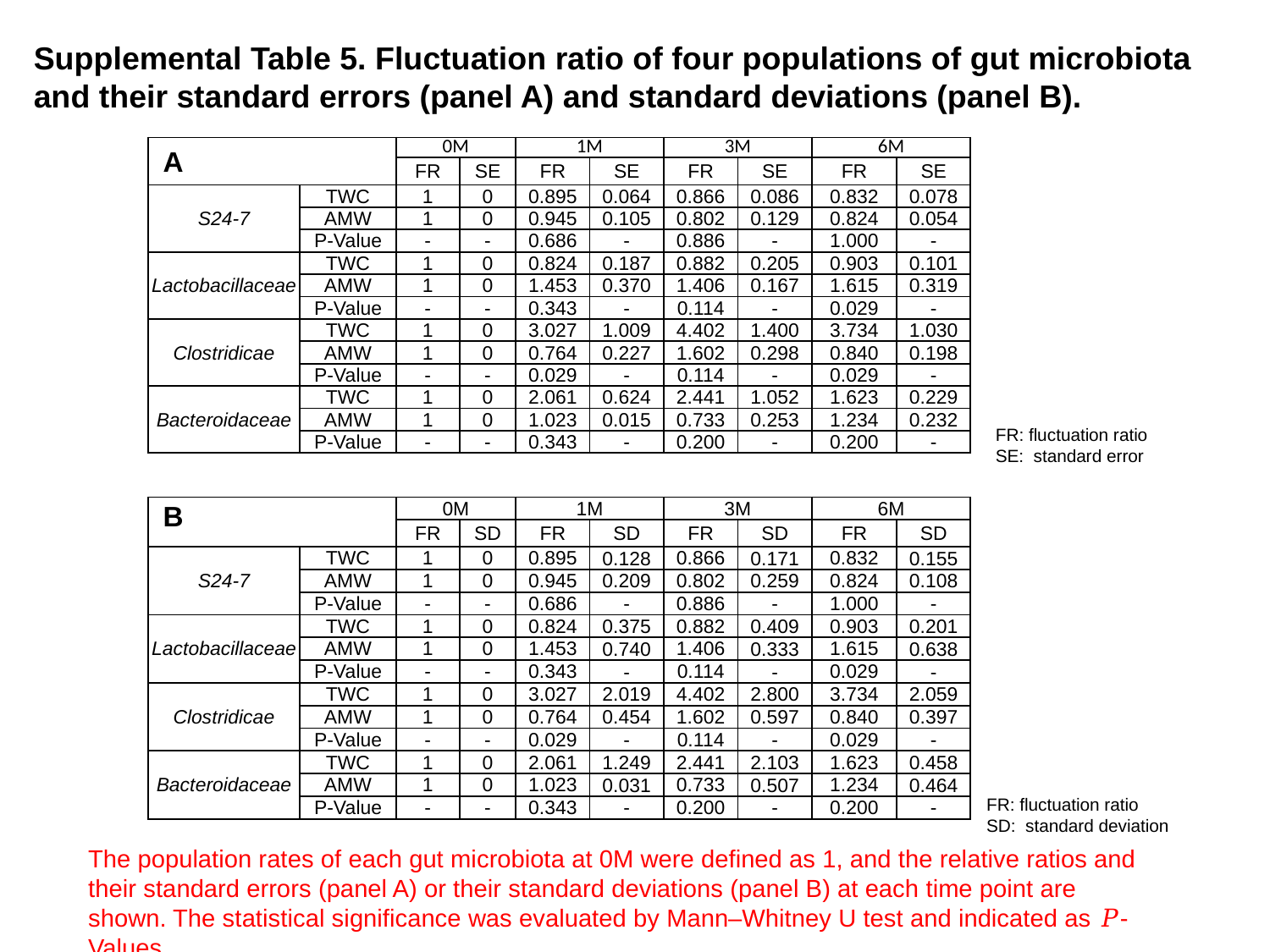

Supplemental Table 5. Fluctuation ratio of four populations of gut microbiota and their standard errors (panel A) and standard deviations (panel B).
| | | 0M | | 1M | | 3M | | 6M | |
| --- | --- | --- | --- | --- | --- | --- | --- | --- | --- |
| | | FR | SE | FR | SE | FR | SE | FR | SE |
| S24-7 | TWC | 1 | 0 | 0.895 | 0.064 | 0.866 | 0.086 | 0.832 | 0.078 |
| | AMW | 1 | 0 | 0.945 | 0.105 | 0.802 | 0.129 | 0.824 | 0.054 |
| | P-Value | - | - | 0.686 | - | 0.886 | - | 1.000 | - |
| Lactobacillaceae | TWC | 1 | 0 | 0.824 | 0.187 | 0.882 | 0.205 | 0.903 | 0.101 |
| | AMW | 1 | 0 | 1.453 | 0.370 | 1.406 | 0.167 | 1.615 | 0.319 |
| | P-Value | - | - | 0.343 | - | 0.114 | - | 0.029 | - |
| Clostridicae | TWC | 1 | 0 | 3.027 | 1.009 | 4.402 | 1.400 | 3.734 | 1.030 |
| | AMW | 1 | 0 | 0.764 | 0.227 | 1.602 | 0.298 | 0.840 | 0.198 |
| | P-Value | - | - | 0.029 | - | 0.114 | - | 0.029 | - |
| Bacteroidaceae | TWC | 1 | 0 | 2.061 | 0.624 | 2.441 | 1.052 | 1.623 | 0.229 |
| | AMW | 1 | 0 | 1.023 | 0.015 | 0.733 | 0.253 | 1.234 | 0.232 |
| | P-Value | - | - | 0.343 | - | 0.200 | - | 0.200 | - |
A
FR: fluctuation ratio
SE: standard error
B
| | | 0M | | 1M | | 3M | | 6M | |
| --- | --- | --- | --- | --- | --- | --- | --- | --- | --- |
| | | FR | SD | FR | SD | FR | SD | FR | SD |
| S24-7 | TWC | 1 | 0 | 0.895 | 0.128 | 0.866 | 0.171 | 0.832 | 0.155 |
| | AMW | 1 | 0 | 0.945 | 0.209 | 0.802 | 0.259 | 0.824 | 0.108 |
| | P-Value | - | - | 0.686 | - | 0.886 | - | 1.000 | - |
| Lactobacillaceae | TWC | 1 | 0 | 0.824 | 0.375 | 0.882 | 0.409 | 0.903 | 0.201 |
| | AMW | 1 | 0 | 1.453 | 0.740 | 1.406 | 0.333 | 1.615 | 0.638 |
| | P-Value | - | - | 0.343 | - | 0.114 | - | 0.029 | - |
| Clostridicae | TWC | 1 | 0 | 3.027 | 2.019 | 4.402 | 2.800 | 3.734 | 2.059 |
| | AMW | 1 | 0 | 0.764 | 0.454 | 1.602 | 0.597 | 0.840 | 0.397 |
| | P-Value | - | - | 0.029 | - | 0.114 | - | 0.029 | - |
| Bacteroidaceae | TWC | 1 | 0 | 2.061 | 1.249 | 2.441 | 2.103 | 1.623 | 0.458 |
| | AMW | 1 | 0 | 1.023 | 0.031 | 0.733 | 0.507 | 1.234 | 0.464 |
| | P-Value | - | - | 0.343 | - | 0.200 | - | 0.200 | - |
FR: fluctuation ratio
SD: standard deviation
The population rates of each gut microbiota at 0M were defined as 1, and the relative ratios and their standard errors (panel A) or their standard deviations (panel B) at each time point are shown. The statistical significance was evaluated by Mann–Whitney U test and indicated as 𝑃-Values.
